# Controllability analysis of molecular pathways points to proteins that control the entire interaction network

**DOI:** 10.1038/s41598-020-59717-6

**Published:** 2020-02-19

**Authors:** Prajwal Devkota, Stefan Wuchty

**Affiliations:** 1 Department of Computer Science, University of Miami, Coral Gables, FL USA; 2 Department of Biology, University of Miami, Coral Gables, FL 33146 USA; 3 Miami Institute of Data Science and Computing, University of Miami, Coral Gables, FL 33146 USA; 4 Sylvester Comprehensive Cancer Center, University of Miami, Miami, FL 33136 USA

**Keywords:** Network topology, Regulatory networks

## Abstract

Inputs to molecular pathways that are the backbone of cellular activity drive the cell to certain outcomes and phenotypes. Here, we investigated proteins that topologically controlled different human pathways represented as independent molecular interaction networks, suggesting that a minority of proteins control a high number of pathways and *vice versa*. Transcending different topological levels, proteins that controlled a large number of pathways also controlled a network of interactions when all pathways were combined. Furthermore, control proteins that were robust when interactions were rewired or inverted also increasingly controlled an increasing number of pathways. As for functional characteristics, such control proteins were enriched with regulatory and signaling genes, disease genes and drug targets. Focusing on evolutionary characteristics, proteins that controlled different pathways had a penchant to be evolutionarily conserved as equal counterparts in other organisms, indicating the fundamental role that control analysis of pathways plays for our understanding of regulation, disease and evolution.

## Introduction

Modern network research recently started to focus on the development of different methods to find nodes that control entire or parts of networks^1–5^. Nodes that allow the topological control of underlying biological networks were found important for different cellular processes^2–4^. A recent analysis of a directed protein-protein interaction network indicated the presence of control proteins that were enriched with disease genes and drug targets as well as carried genomic alterations in diverse cancer types^2^. While these results were found in a single interaction network, the inner workings of a cell are usually organized through an elaborate network of distinct molecular pathways. In particular, each pathway is represented as a network of directed molecular interactions that provide a certain cellular function. As a consequence of the representation of pathways as directed networks, we surmised that pathway-specific proteins may allow the control of a given pathway. As pathway crosstalk is established through proteins that appear in more than one pathway, we hypothesized that sets of proteins may exist, controlling many different pathways at the same time. As a consequence, proteins that control many different pathways may mediate functional, biomedical and evolutionary significance, indicating *e.g*. disease or essential genes.

To fill this knowledge gap, we determined and analyzed proteins that structurally controlled pathways, represented as separate directed networks of interactions between proteins. Notably, we found that a small minority of proteins controlled a high number of pathways and *vice versa*. Transcending different topological levels, proteins that increasingly controlled pathways also appeared as control proteins in a combined pathway network that we obtained by pooling interactions from all underlying pathways. Furthermore, proteins that controlled a large number of pathways appeared to be resilient to rewiring and flipping the direction of interactions. Strongly indicating their biological significance, control proteins were enriched with regulatory and signaling genes, disease genes and drug targets on both topological levels. Anticipating that such topological features may carry an evolutionary blueprint we also observed that proteins that controlled different pathways had a penchant to be evolutionarily conserved as equal control counterparts in other organisms.

## Results

**Figure 1:**
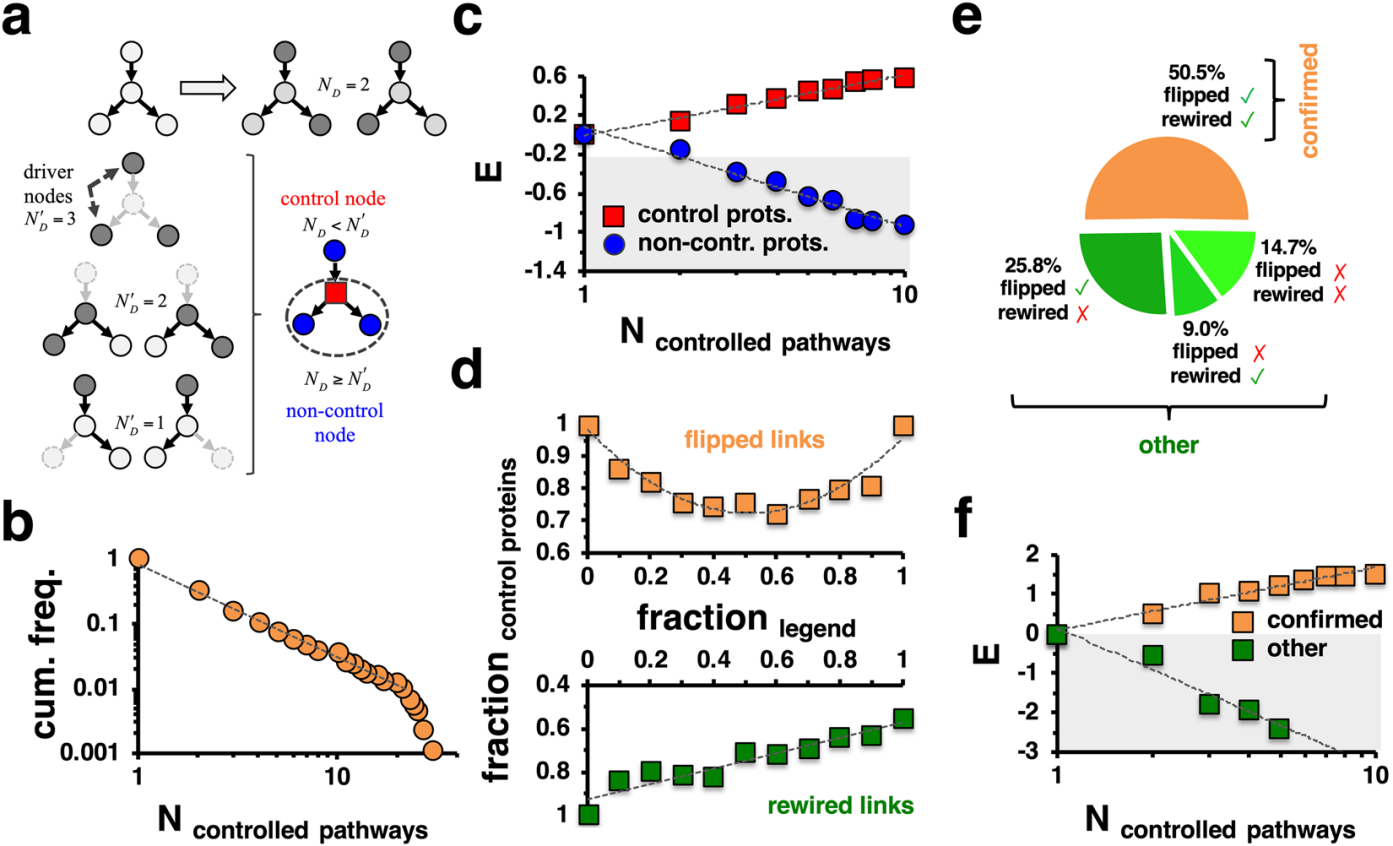
Topological characteristics of pathway controlling proteins. **(a)** In the schematic representation of the controllability framework the application of a maximum matching algorithm allows the determination of ${N}_{D}=2$ driver nodes. To find control nodes, we separately eliminated each node and determined the number of driver nodes ${N{\prime} }_{D}$ in the network thus obtained. We found a control node if the elimination of a node increased the number of driver nodes compared to the unperturbed network, ${N{\prime} }_{D} > {N}_{D}$. **(b)** Utilizing 276 KEGG pathways, we found that the cumulative frequency distribution of the number of pathways that proteins controlled followed a power-law, suggesting that a minority of proteins controls a large number of pathways and *vice versa*. **(c)** After determining control nodes in a network of 67,038 directed interactions between 5,398 proteins that we obtained by combining interactions of all pathways we randomly sampled such sets of control proteins. Notably, control proteins in the combined interaction network were enriched in groups of proteins that controlled an increasing number of pathways. **(d)** In the combined network we flipped and rewired given fractions of interactions. Notably, flipping the direction of roughly half of all interactions limited our ability to confirm control proteins the most. In turn, rewiring interactions continuously decreased the fraction of confirmed nodes. **(e)** When we flipped 50% of all interactions and rewired all interactions, respectively, half of all control proteins were confirmed. **(f)** More quantitatively, we randomly sampled sets of control proteins that were confirmed after flipping and rewiring interactions and found that such proteins were enriched in groups of proteins that controlled an increasing number of pathways. In turn, the set of remaining proteins was found strongly diluted in groups of proteins that controlled a limited number of pathways.

As the majority of pathway specific interactions were directed, indicating flow of biological information from *e.g*. a transcription factor to an expressed gene, we utilized 276 human KEGG^6^ pathways that had at least 5 directed interactions. In each pathway, we mapped directed interactions to a bipartite graph, where partitions referred to proteins that started and ended direct interactions. To find potential control proteins, we determined the largest subset of interactions in each pathway network called a maximum matching, where no two interactions shared a common start and end point. Unmatched nodes that correspond to any maximum matching were previously shown that they can be chosen as driver nodes to structurally control the whole underlying network^1^. To further assess the relevance of nodes we considered the topological consequences of their removal. In particular, we defined a node as a control node, if the total number of driver nodes increased in a maximum matching after its removal^2^. Such nodes are considered important for the control of the underlying network as more driver nodes emerged as a consequence of their removal (Fig. 1a). Furthermore, we considered nodes that keep the number of driver nodes constant (*i.e*. neutral) or decreased the number of driver nodes (*i.e*. dispensable^2^) upon their deletion as irrelevant for control. Determining such control proteins in each pathway specific network separately, we counted the number of pathways that a protein controlled. Notably, we observed that the corresponding frequency distribution followed a power-law like decay (Fig. 1b), indicating that a small number of proteins controlled a high number of pathways and *vice versa*. To show the independence of our results from the underlying pathway data, we determined control proteins in 1,192 human Reactome^7^ pathways and corroborated our initial finding (Supplementary Fig. 1a). Furthermore, we hypothesized that proteins that controlled an increasing number of different pathways separately may also appear as control proteins in a network that was composed of all pathway interactions. Pooling all 276 KEGG pathways, we obtained a network of 67,038 directed interactions between 5,398 proteins and found 577 (10.7%) control proteins. More quantitatively, we randomly sampled such a set of control proteins in the combined pathway network and determined their enrichment in bins of proteins that controlled an increasing number of KEGG pathways. In Fig. 1c, we observed that these proteins were enriched in groups of proteins that controlled an increasing number of pathways while non-control proteins appeared diluted. To establish the independence of our results from the choice of pathway data, we pooled all 1,192 Reactome pathway specific networks, obtaining a directed network of 180,020 edges between 8,084 proteins. Randomly sampling all 941 (11.6%) control proteins we observed similar results in the pooled network of interactions in Reactome pathways (Supplementary Fig. 1b). Considering the combined networks of KEGG pathways, we determined the degree of corresponding proteins, indicating that both (non-)control proteins had fat tails (Supplementary Fig. 2a). Such observations translated into a propensity of control proteins to be enriched in bins of strongly interacting proteins while we found no enrichment/dilution signals when we considered non-control proteins (Supplementary Fig. 2b). Such observations were corroborated when we considered a combined network of Reactome pathways (Supplementary Fig. 2c,d).

In a robustness analysis, we flipped the direction of given fractions of interactions in the combined pathway network and determined the number of control nodes in a network thus obtained (upper panel, Fig. 1d). Corroborating earlier results in a network of directed protein-protein interactions^2^, flipping half of all interactions corresponded  to the lowest fraction of control nodes that were found in the original, unperturbed network. Furthermore, we rewired a given fraction of directed interactions keeping the underlying degree distributions of nodes in the unperturbed network, indicating that half of all control nodes were robust toward the complete rewiring of the network (lower panel, Fig. 1d). To paint a coherent picture of the robustness of control proteins, we determined control nodes in a network where we flipped one half of all directed interactions. Furthermore, we determined control nodes in a network where we rewired all interactions, keeping the degree distributions of the underlying nodes. Fig. 1e indicates that 50.5% of all control nodes in the underlying unperturbed network were robust in the presence of rewired and flipped interactions. More quantitatively, we randomly sampled sets of control proteins that were confirmed after flipping and rewiring interactions in Fig. 1f. Notably, such robust control proteins were almost entirely found enriched in bins of proteins that controlled a large number of pathways. To corroborate our results, we repeated this analysis using the combined network of Reactome pathways and observed similar results (Supplementary Fig. 3a–c).

**Figure 2:**
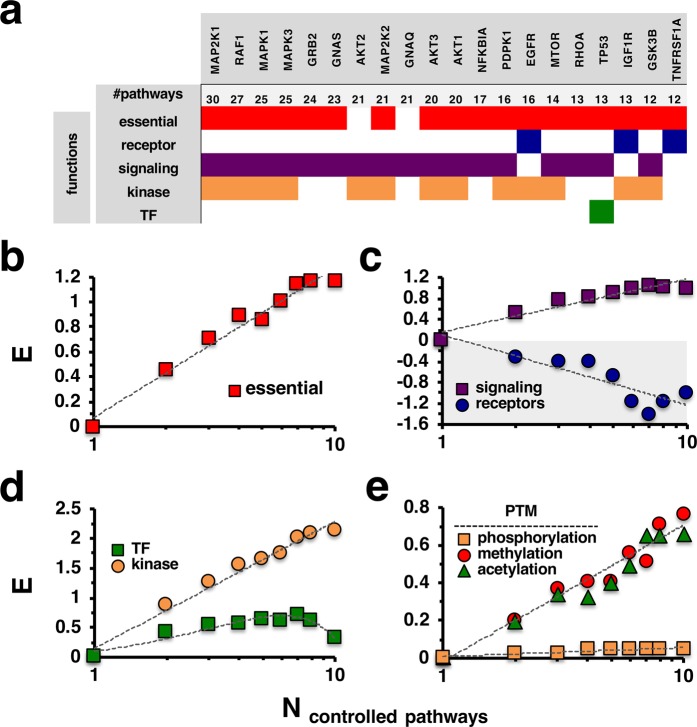
Functional characteristics of pathway controlling proteins. **(a)** We collected 20 proteins that controlled the highest number of pathways. We observed that such proteins were mostly essential and had kinase but rarely transcription factor functions. While sporadically membrane-bound receptors, the majority of such proteins were involved in signaling activities. More quantitatively, we randomly sampled sets of control proteins and found that proteins that controlled an increasing number of pathways were enriched with **(b)** essential genes and **(c)** signaling functions, while they rarely were membrane-bound receptors. **(d)** Such proteins were more frequently enriched with kinases than transcription factors. **(e)** As for post-translational modifications, proteins that controlled an increasing number of pathways were strongly enriched with acetylated and methylated proteins while we only found a modest enrichment of phosphorylated proteins.

On a functional level, we presented the 20 most KEGG pathway-controlling proteins in the table of Fig. 2a and observed that they were frequently essential for the survival of the cell. While these proteins were hardly transcription factors and membrane bound receptors, they also frequently carried kinase and signaling functions when we excluded membrane bound proteins (Fig. 2a). On a more quantitative level, we randomly sampled 2,708 essential human genes^8,9^ that we found enriched among proteins that controlled an increasing number of pathways (Fig. 2b). Focusing on control proteins in the combined pathway network, we found that essential genes were significantly enriched as well, while they appeared diluted in the set of remaining proteins (Supplementary Fig. 4a). Considering 4,408 proteins that were involved in signaling functions (excluding membrane bound proteins), we found that such proteins appeared enriched, while 5,701 receptor proteins were found to be diluted among proteins that controlled an increasing number of pathways (Fig. 2c), results that we corroborated in the combined network (Supplementary Fig. 4b). As a corollary of our observation that control proteins are enriched with signaling functions, we hypothesized that these proteins may be significantly involved in regulatory processes. Considering a set of 1,471 manually curated sequence-specific DNA-binding transcription factors^10,11^ and 501 kinases^12^, we found that proteins that control an increasing number of different pathways were more frequently enriched with kinases than transcription factors (Fig. 2d), results that we confirmed in the combined pathway network as well (Supplementary Fig. 4c). In turn, proteins that received post-translational modifications as a consequence of regulatory activity may be important for pathway control. Randomizing sets of methylated, acetylated and phosphorylated proteins we observed that methylated and acetylated proteins increasingly appeared in sets of proteins that controlled an elevated number of pathways while phosphorylated target appeared significantly less enriched (Fig. 2e). Still, all targets of posttranslational modifications appeared significantly enriched in the set of proteins that controlled the combined pathway network (Supplementary Fig. 4d). To confirm that our results were independent of KEGG pathway data, we used Reactome pathway data, indicating similar results when we considered proteins that controlled single pathways (Supplementary Fig. 5) as well as a network of all pathway interactions combined (Supplementary Fig. 6**)**.

**Figure 3:**
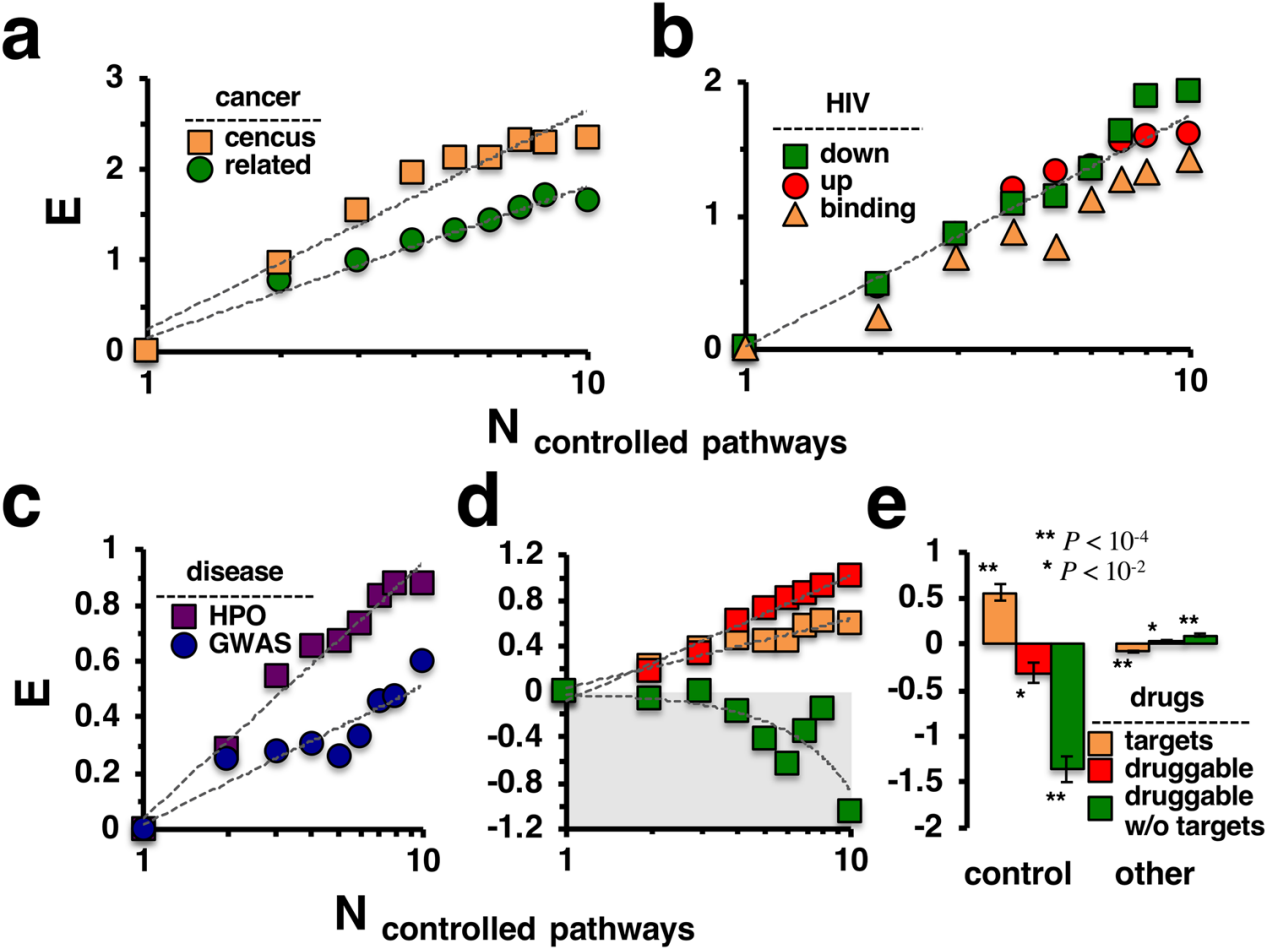
Pathway controlling proteins were enriched with disease genes and drug targets. **(a)** Using a compilation of census cancer genes and a set of onco- and tumor-suppressor genes, we found that such cancer related genes strongly appeared in groups of proteins that controlled an increasing number of pathways. **(b)** Such control protein groups were enriched with targets that the HIV virus binds as well as genes that were dys-regulated after viral infection. **(c)** More generally, disease genes from genetic (HPO) and genomic (GWAS) sources were enriched in groups of proteins that frequently controlled pathways. **(d)** FDA approved drug targets and druggable genes were enriched in such groups of control proteins as well. However, a subset of druggable genes that excluded known drug targets appeared diluted. **(e)** In turn, approved drug targets were enriched in the set of proteins that controlled the network of the combined pathway network while druggable genes in general however appeared diluted.

As a corollary of our previous results, we hypothesized that control proteins may play a role for the transition between healthy and disease conditions. Considering a set of 568 genes causally implicated in oncogenesis as annotated by the Sanger Center^13^, we found that enrichment of cancer genes increased as proteins controlled more pathways (Fig. 3a), a result that was confirmed in the combined KEGG pathway network (Supplementary Fig. 7a). Furthermore, we considered a set of 1,259 onco- and tumor-suppressor genes that were predicted to be cancer-related^14^ and obtained similar results, further substantiating our observations (Fig. 3a, Supplementary Fig. 7a). As for viral infections, we analyzed a set of 544 human proteins that physically interacted with proteins of the HIV virus^15^ as well as 788 human genes that were down-regulated and 1,118 genes that were up-regulated upon HIV infection^15^. Notably, such infection relevant genes appeared enriched in groups of proteins that controlled an increasing number of pathways (Fig. 3b), a result that was corroborated with proteins that controlled the combined pathway network (Supplementary Fig. 7b). As for genetic causes of diseases, we further considered a set of 2,661 genes that carried disease-causing mutations^16,17^. We found that such disease genes were enriched among control proteins in an increasing number of pathways (Fig. 3c). Considering a set of 11,002 disease genes as identified by GWAS studies^18^, we were able to corroborate this result. Furthermore, we obtained similar results when we analyzed the enrichment of such genes in the set of control proteins in the combined pathway network (Supplementary Fig. 7c). Notably, enrichment levels of GWAS related genes were generally lower than genes that harbored disease-causing mutations. Investigating the transformation of a disease to a healthy state, we utilized a set of 2,289 Food and Drug Administration (FDA) approved drug targets^19^. Notably, drug targets predominantly appeared in groups of proteins that controlled an increasing number of pathways (Fig. 3d**)**. We also considered a set of druggable proteins as they carried protein folds favoring interactions with chemical compounds for our enrichment analysis. Generally, we found that drug targets were enriched in proteins that controlled many pathways. However, genes that were druggable but not approved drug targets appeared to be diluted. While the enrichment of drug targets in the set of proteins that controlled the combined pathway network was confirmed we found the opposite for both sets of druggable genes that mostly appeared among non-control proteins (Fig. 3e). To corroborate the independence of these observations, we obtained similar results with Reactome pathway data when we considered the enrichment of disease genes and drug targets among proteins that controlled a large number of pathways (Supplementary Fig. 8) as well as the combined pathway interaction network (Supplementary Fig. 9**)**.

**Figure 4:**
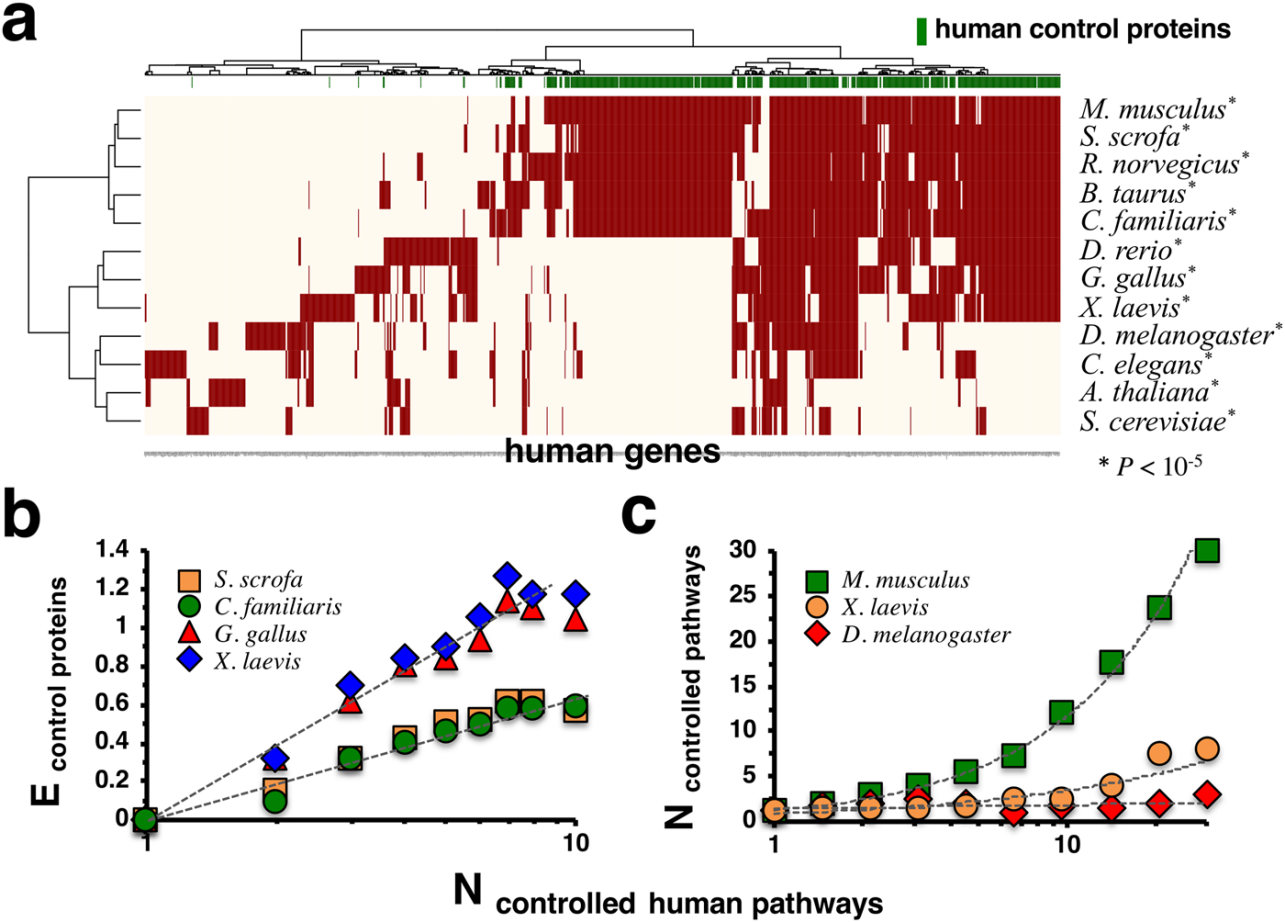
Evolutionary conservation of pathway controlling proteins. **(a)** We mapped human genes to conserved proteins in different organisms that controlled networks of interactions when we combined all pathway interactions in the underlying organism. Notably, we observed that human control proteins were significantly enriched with corresponding control proteins in other organisms (P < 10^−5^, Fisher’s exact test). **(b)** Human proteins that controlled an increasing number of pathways appeared enriched with evolutionarily conserved proteins that controlled networks of combined pathways in other organisms. **(c)** As a corollary, we found that the numbers of pathways human proteins controlled correlated well with their organism specific counterparts.

Based on the obtained results so far, we further assumed that the topological and biological role of control proteins may be reflected by their propensity to be evolutionarily conserved. In particular, we labeled all proteins in different organisms that had a human ortholog based on KEGG orthology groups. In the heatmap in Supplementary Fig. 10 we however found that proteins that controlled the combined KEGG human pathway network appeared randomly scattered among orthologous proteins in closely related organisms. Constructing directed interaction networks by combining all KEGG pathways of a given organism, we determined control proteins in these combined organism specific networks. In the heatmap in Fig. 4a we labeled all human proteins with conserved control proteins in closely related organisms. Notably, human control proteins in the combined pathway network significantly aligned with their conserved counterparts in different organisms, an observation that we quantitatively confirmed by a Fisher’s exact test (P < 10^−5^). Extending such considerations, we observed that proteins that controlled an increasing number of human pathways were preferably conserved as proteins that controlled combined pathway networks in different organisms (Fig. 4b). Notably, enrichment levels differed between *S. scrofa* and *C. familiaris* and more distantly related organisms such as *G. gallus* and *X. laevis*. As a corollary, we hypothesized that human proteins that control an increasing number of pathways have conserved counterparts that control a similar number of pathways in other organisms. Determining control proteins in pathways of different organism separately, we indeed found that the number of pathways that proteins controlled in different organisms correlated well with their human counterparts, indicating different levels of evolutionary kinship (Fig. 4c).

## Discussion

In this work, we determined proteins that control pathways represented as separate, unweighted, directed networks of directed molecular interactions. In such networks, structural controllability of linear dynamics is secured by a set of driver nodes^1^. However, we deviated from the original concept of structural controllability as we used a heuristic to assess the influence of each node on the cardinality of the set of driver nodes when a node in question is deleted. As driver nodes need to be tweaked to push the network to any given dynamic state, the deletion of a node that increased the number of driver nodes in the perturbed network exacerbates the control of the unperturbed network and *vice versa*. In particular, we considered a node in the underlying networks as relevant for the control of the underlying network if the number of driver nodes increased as a consequence of the deletion of a given node^2^. If the number of driver nodes decreased (*i.e*. a redundant node in^2^) or remained unchanged upon deletion of a node (*i.e*. a neutral node in^2^), we considered the deleted node as irrelevant for control. As a consequence, a node that initially was found to be a driver node does not necessarily translate into a control node, as its deletion may lower the number of driver nodes or be compensated by a newly emerging driver node in the perturbed network. In turn, a node that initially did not participate in the set of driver nodes, may increase the number of driver nodes upon its deletion, suggesting that it is relevant for controlling the underlying network. While sets of driver nodes are usually diluted with highly connected nodes, we found that control nodes that increased the number of driver nodes upon their deletion were enriched with strongly connected nodes. In turn, we observed that nodes, that kept the number of driver nodes at least unchanged upon their deletion, did not show any propensity to be enriched/diluted in bins of increasingly connected nodes. While corroborating previous results in directed networks^2^ such observations are also consistent with previous investigations in undirected, unweighted protein interaction networks, where proteins that were relevant for the control of the underlying networks were preferentially well connected^20,21^. In comparison to approaches that determine minimum dominating sets^22^, we stress that the current approach more realistically assumes that links of driver nodes are controlled at the same time^1^. While such results emphasize the role of highly connected nodes (*i.e*. hubs), we stress that a large degree alone is not necessarily a criterion that qualifies a protein to be a control node as the degree distributions of both control and non-control proteins have fat tails, indicating a small minority of highly connected nodes. Furthermore, the definition of hubs depends on arbitrarily set degree thresholds, capturing the local vicinity of a node. In turn, the way to determine control proteins accounts for the whole network, providing an optimal set of strategically placed proteins without the need of arbitrary parameters.

Notably, the frequency distribution of the number of pathways that were controlled by given proteins decayed as a power-law, suggesting a minority of proteins that controlled many pathways and *vice versa*. Such a characteristic is rooted in the propensity of pathways to substantially overlap, as pathways share genes, allowing pathways to cross-talk. Furthermore, we found that proteins that controlled many different pathways separately also had a heightened chance to control a network of interactions obtained by pooling all pathway interactions. In a similar vein, control nodes that remained robust in networks where we flipped and rewired interactions were preferably enriched among control nodes in an increasing number of pathways. Such observations suggest that the combined pathway network still carried the blueprint of the underlying pathways, transcending different levels of topological organization despite omitting their boundaries.

As such results highlighted the topological placement of control proteins, the question remained if these characteristics translated into a governing, meaningful biological role. Emphasizing their biological importance, control proteins on both topological levels were enriched with essential genes. While signaling proteins were found enriched as well, membrane-bound receptor proteins appeared to be diluted. Such results indicated that pathways were rather controlled by proteins deeply embedded in signaling pathways than by their entry points. Furthermore, topological placement of control proteins may also support functional interactions that exert biological control. Indeed, kinases were strongly enriched in a set of proteins that controlled an increasing number of pathways. Moreover, we observed a lower enrichment of transcription factors, indicating their ubiquitous presence in terms of gene regulation. In turn, kinases may control a large number of pathways to collect and disseminate biological information. Further, we expected that recipients of post-translational modifications may be control proteins as well. Indeed, we found that acetylated and methylated substrates were strongly enriched in the sets of proteins that controlled an increasing number of pathways. While still enriched, we found much weaker signals when we considered phosphorylated proteins. The latter observation may be a consequence of the fact that a considerable amount of known pathways cover signaling functions that strongly feature phosphorylation events. Nonetheless, the prevalence of kinases and proteins with posttranslational modifications suggested that the placement of control proteins in different pathways was crucial for the dissemination of biological information.

In terms of network medicine, disease causing mutations exert their influence through interactions of afflicted protein^23^. As a consequence, we expected that proteins that controlled an increasing number of pathways may be enriched with disease genes as the placement of control proteins allows for fast transmission. Indeed, disease genes that carried mutations were strongly enriched in sets of control proteins. Surprisingly, we observed that disease genes that were identified from genome-wide association studies were less strongly enriched. Such an observation may be rooted in the fact that GWAS studies rather identify non-coding genomic regions but not specific disease causing genes^2^. As a corollary, we corroborated the role of control proteins as central to the dissemination of biological information to transform a cell from a disease to a healthy state, when we investigated the enrichment of drug targets. While we found that drug targets and druggable genes were enriched with proteins that controlled a large number of pathways, we observed the opposite when we considered druggable genes that were not approved by FDA as drug targets. Considering control proteins in the pooled pathway network, we surprisingly found that only approved drug targets were enriched, suggesting that protein domain-folds that can interact with drugs alone are putatively no good indicators of a potential drug target.

As a final consideration, we expected that the topological and biological relevance of proteins that controlled pathways was an evolutionarily conserved feature. Initially, we surprisingly found that control proteins in the human combined pathway network did not appear as particularly conserved in other organisms. Yet, we observed a strong conservation signal, when we considered ortholog proteins that controlled the combined pathway network in different organisms. Transcending different topological levels, human proteins that controlled an increasing number of pathways had conserved counterparts in organism-specific combined pathway networks that well reflected evolutionary distance by corresponding enrichment levels. In particular, increasing evolutionary distance to human was reflected by a decreasing pool of orthologs that may translate into lower enrichment of human control proteins. Furthermore, we observed strong correlations between the number of controlled pathways, when we compared human control proteins to their conserved counterparts in other organisms. Although pathways in other organisms are mostly inferred through the aid of orthologous proteins, our results still suggested that the evolution of pathways retained topological control features as well.

## Materials and Methods

### Pathway information

We collected interaction information of pathways in different organisms from the KEGG^6^ and Reactome^7^ databases as parsed with the graphite R tool^24^. The vast majority of annotated interactions in these pathways were directed, indicating flow of biological information from *e.g*. a kinase to a substrate. Furthermore, physical interactions between proteins (such as protein-protein interactions) were annotated as undirected. We only accounted for directed interactions and considered pathways with at least 5 directed interactions, resulting in 276 KEGG and 1,192 Reactome pathway specific networks. Based on these data sources, all interactions of pathways were pooled to obtain a directed network of 67,038 interactions between 5,398 proteins using KEGG pathways and 8,084 proteins in 180,020 edges using Reactome pathways.

### Functional sets of genes

We obtained 2,708 human essential genes from the online gene essentiality database (OGEE)^8^ and the Database of Essential genes (DEG)^9^. We collected a set of 1,471 manually curated sequence-specific DNA-binding human transcription factors from^10,11^ and 501 human kinases from the Kinome NetworkX database^12^ which curates kinase information from literature and other databases. As for posttranslational modifications (PTM) we used 17,511 phosphorylated proteins, 6,928 acetylated proteins and 5,418 methylated proteins from the PhosphoSitePlus database^25^. For signaling genes, we used 4,408 genes that were annotated with a signaling function without receptor domain function from Gene Ontology (GO)^26^. Furthermore, we used 5,701 genes that carried a trans-membrane protein domain^27^.

### Disease genes and drug targets

As representative sets of cancer genes, we used 568 genes that were annotated by the Sanger Center as causally implicated in oncogenesis^13^ as well as 1,259 onco- and tumorsuppressor genes that were predicted as cancer-related^14^. As for viral infections, we utilized 544 human proteins that interacted with proteins of the HIV virus from the Human Immunodeficiency Virus Type 1 (HIV-1) Human Interaction Database^15^. From the same source we used 788 human genes that were down-regulated and 1,118 genes that were up-regulated upon HIV infection.

As for disease genes, we accounted for 2,661 genes that were identified as causal for a disease as of human phenotype ontology database (HPO)^16^ that is based on the Online Mendelian Inheritance in Man (OMIM) database^17^. Furthermore, we collected 11,002 disease genes that were identified from GWAS studies^18^.

As for drug targets, we used a set of 2,289 drug targets that were approved by the Food and Drug Administration (FDA) as of the DrugBank database^19^. Furthermore, we accounted for 2,436 genes that were annotated as druggable as these proteins carried domains that were deemed suitable to interact with drugs^28^.

### Controllability analysis

Driver nodes were determined in pathways that were represented as directed un-weighted networks of interactions between genes in the underlying pathways. Such drivers are defined as nodes that are sufficient to ensure the structural controllability of linear dynamics^1^. In particular, such a structural controllability problem can be mapped to a maximum matching problem, assuming that a network of direct interactions is a graph-based proxy of the underlying dynamical system. The maximum matching problem can be solved in polynomial time by the Hopcroft-Karp algorithm^29^, mapping a directed to a bipartite network. Specifically, we mapped directed links to edges between partitions of nodes that start and end edges. In the matching, a subset of edges *M* is a matching of maximum cardinality in a directed network if no two edges in *M* share a common starting and ending vertex. Vertices that do not appear in *M* are unmatched and have been shown to be nodes that structurally control the underlying network^1^. As a corollary, a maximum matching implies the presence of a minimum set of such driver nodes of size *N*_*D*_.

To assess the impact of network nodes on the controllability of the underlying directed network we applied the following heuristic^2^ (Fig. 1a): After a node is removed from the underlying network, we determined the size ${{\rm{N}}{\prime} }_{{\rm{D}}}$of driver nodes in the changed network. If ${{\rm{N}}{\prime} }_{{\rm{D}}} > {{\rm{N}}}_{{\rm{D}}}$, the node is classified as indispensable (*i.e*. a control node) if the number of driver nodes increased. In other words, the deletion of a node increased the number of nodes that allow the control the underlying network. If ${{\rm{N}}{\prime} }_{{\rm{D}}}\le {{\rm{N}}}_{{\rm{D}}}$, the node is classified as non-controlling as the number of driver nodes remained unchanged (neutral node) or decreased (dispensable node)^2^.

### Enrichment analysis

In a group *i* of control proteins the corresponding number of proteins with a certain characteristic *A*, ${{\rm{N}}}_{{\rm{i}}}^{{\rm{A}}}$ (e.g. being essential or a drug target) were determined. Randomly sampling a set of proteins with characteristic *A*, we calculated the corresponding random number of control proteins with *A*, ${{\rm{N}}}_{{\rm{i}}}^{{\rm{r}},{\rm{A}}}$. We defined the enrichment of proteins with characteristic *A* in a group *i* of control proteins that appear in a given number of pathways as ${{\rm{E}}}_{{\rm{i}}}^{{\rm{A}}}={{\rm{lg}}}_{2}({{\rm{N}}}_{{\rm{i}}}^{{\rm{A}}}/{{\rm{N}}}_{{\rm{i}}}^{{\rm{r}},{\rm{A}}})$.

Furthermore, the enrichment of proteins with a certain characteristic *A* was determined as a function of the number of pathways *k*, that given proteins control. In particular, ${{\rm{N}}}_{\ge {\rm{k}}}^{{\rm{A}}}$ is the number of proteins with *A* that controlled $\ge k$ pathways. Randomly sampling a set of proteins with characteristic *A*, we calculated the corresponding random number of ${N}_{\ge k}^{r,A}$. The enrichment of these proteins in a group of proteins that control at least *k* pathways was then defined as ${{\rm{E}}}_{\ge {\rm{k}}}^{{\rm{A}}}={{\rm{lg}}}_{2}({{\rm{N}}}_{\ge {\rm{k}}}^{{\rm{A}}}/{{\rm{N}}}_{\ge {\rm{k}}}^{{\rm{r}},{\rm{A}}})$. In both cases, ${{\rm{E}}}_{\{{\rm{i}},\ge {\rm{k}}\}}^{{\rm{A}}} > 0$ points to an enrichment of feature *A* and *vice versa*. In particular, proteins with feature *A* were sampled 10,000 times and averaged enrichment values thus obtained.

## Supplementary information

Supplementary Figures.
