# Supplementary material for: Controllability analysis of molecular pathways points to proteins that control the entire interaction network
Source: Sci Rep. 2020 Feb 19;10:2943. doi: 10.1038/s41598-020-59717-6 (PMC7031241; doi:10.1038/s41598-020-59717-6)
Supplement: Supplementary file 1 — Supplementary Figures. [file 41598_2020_59717_MOESM1_ESM.docx]

**Controllability analysis of molecular pathways points to proteins that control the entire interaction network** – Suppl. Materials

Prajwal Devkota^1^ and Stefan Wuchty^1,2,3,4,*^

^1^ Department of Computer Science, University of Miami, Coral Gables, FL 33146, U.S.A.

^2^ Department of Biology, University of Miami, Coral Gables, FL 33146, U.S.A.

^3^ Miami Institute of Data Science and Computing, University of Miami, Coral Gables, FL 33146, U.S.A.

^4^ Sylvester Comprehensive Cancer Center, University of Miami, Miami, FL 33136, U.S.A.

*corresponding author

Stefan Wuchty

Dept. of Computer Science

Univ. of Miami

1365 Memorial Drive

Coral Gables, FL 33146, USA

e-mail: wuchtys@cs.miami.edu

**
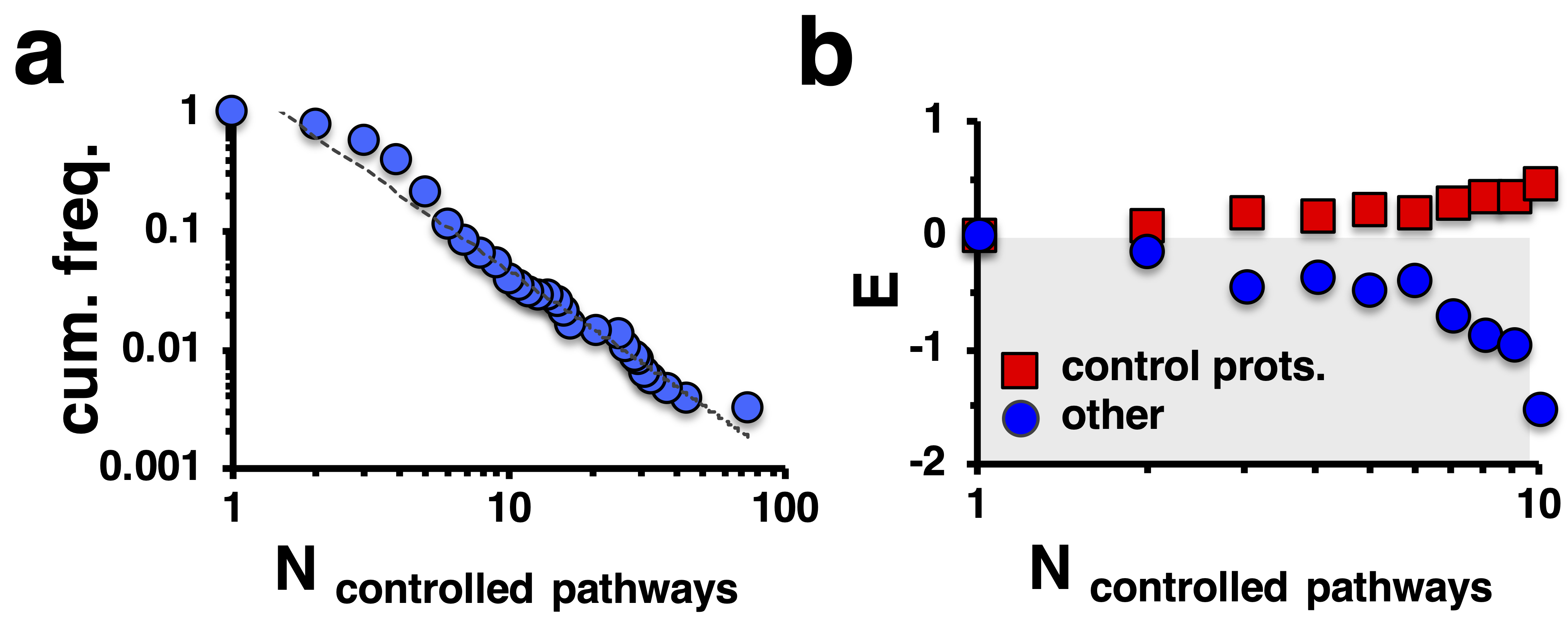
Suppl. Figure 1.** Control proteins in Reactome pathways I. **(a)** The frequency distribution of the number of Reactome pathways that a given protein controls followed a power-law. **(b)** Combining all interactions of Reactome pathways we determined control proteins in such a large interaction network and calculated their enrichment as a function of the number of pathways a protein controls. While control proteins in the pooled network preferably appeared in bins of proteins that control a high number of pathways, remaining proteins were diluted.


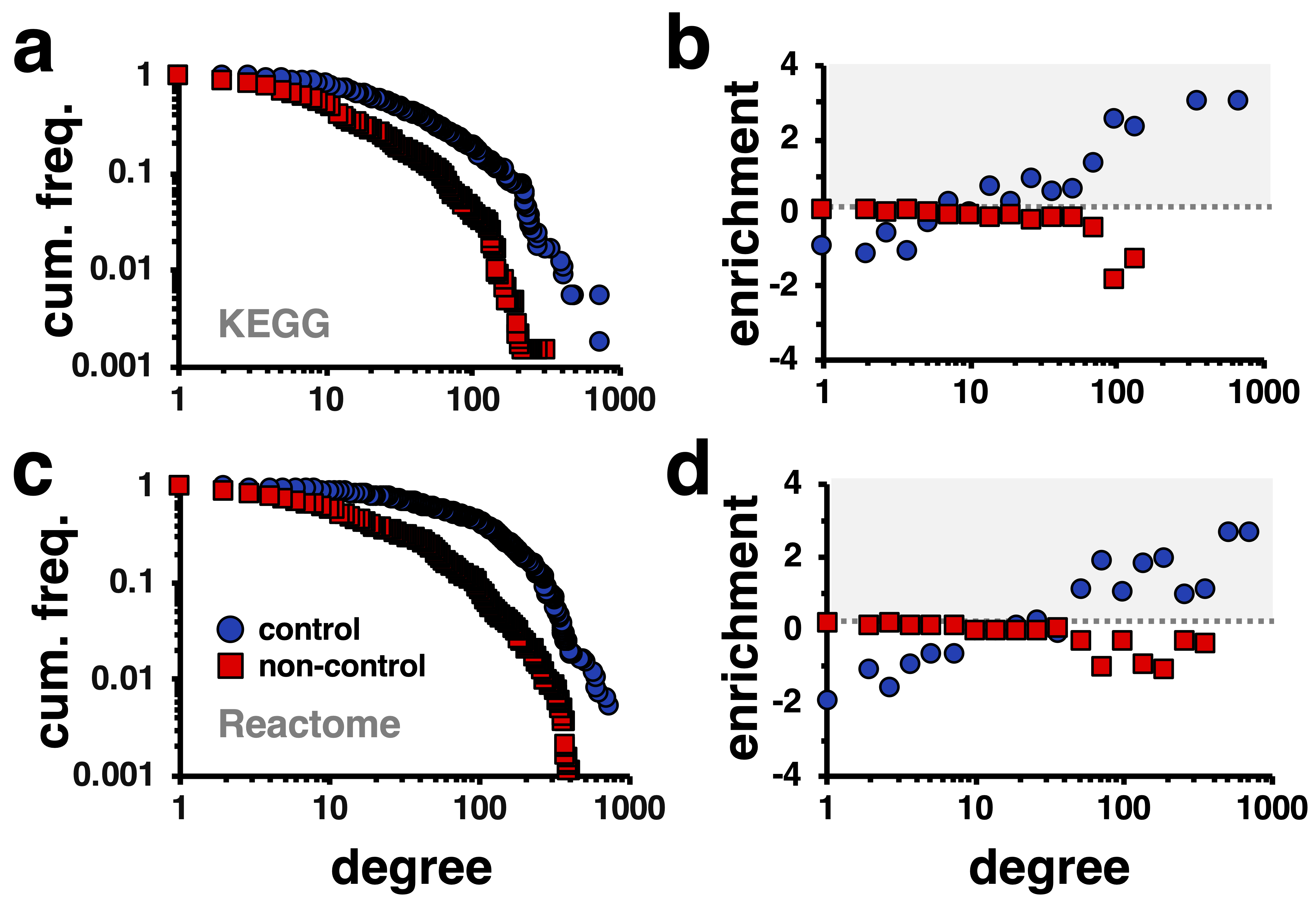


**Suppl. Figure 2.** (non-)control proteins as a function of their connectivity.

**(a)** In the combined network of interactions in Kegg pathways, we observed that degree distributions of (non-)control proteins have fat tails. **(b)** Considering (non-)control proteins in the combined network of interactions in pathways, we observed that control proteins tend to be highly connected. In turn, non-control proteins appeared to be neither enriched nor diluted in bins of higher connected proteins. **(c-d)** We corroborate these findings when we considered a network that combined all Reactome pathways.

**
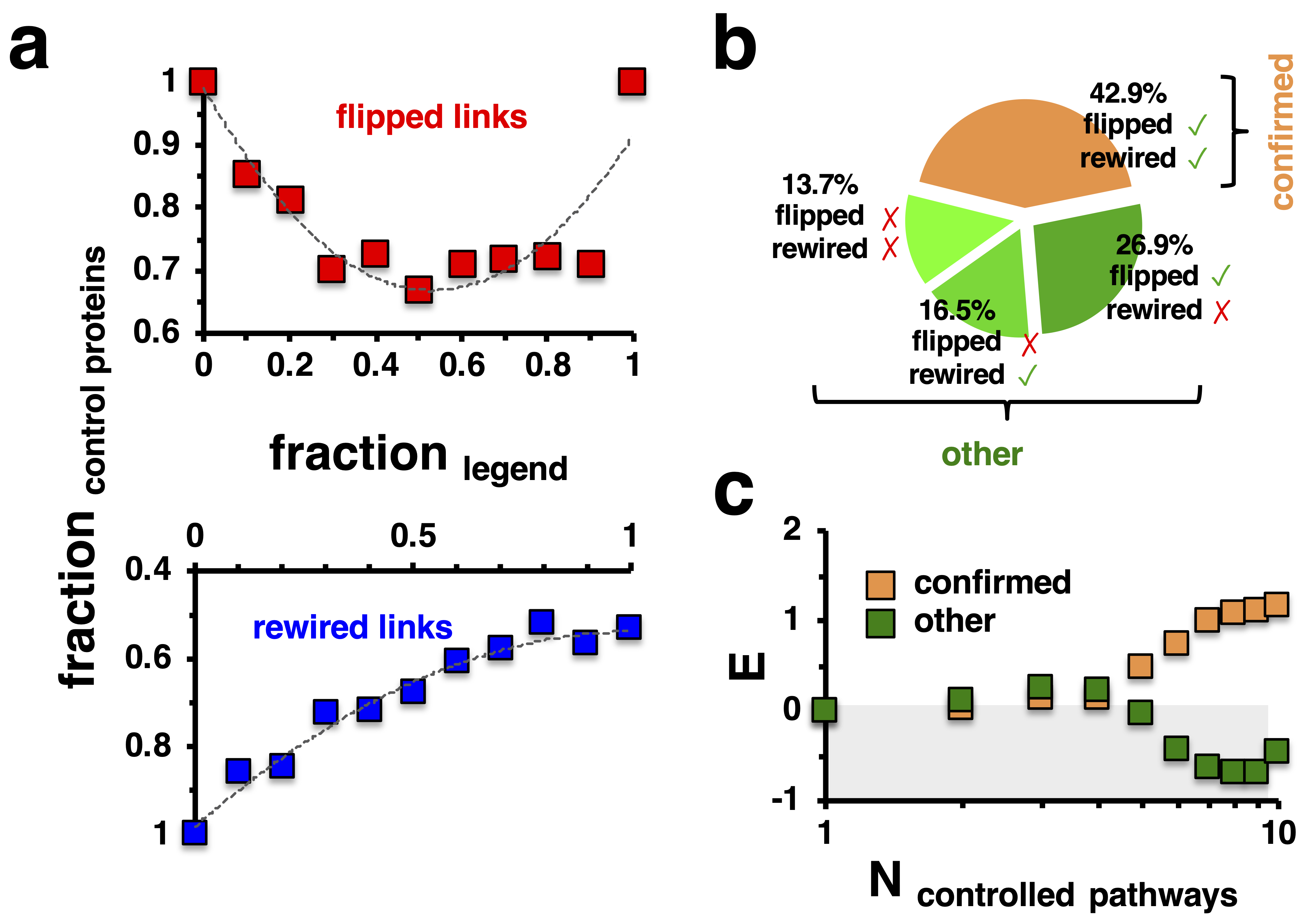
Suppl. Figure 3.** Robustness analysis of control proteins in Reactome pathways. **(a)** In the combined network we flipped and rewired given fractions of interactions. Notably, flipping the direction of roughly half of all interactions limited our ability to confirm control proteins the most. In turn, rewiring interactions continuously decreased the fraction of confirmed nodes. **(b)** When we flipped 50% of all interactions and rewired all interactions, respectively, roughly 42% of all control proteins were confirmed. **(c)** More quantitatively, we randomly sampled sets of control proteins that were confirmed after flipping and rewiring interactions and found that such proteins were enriched in groups of proteins that controlled an increasing number of pathways. In turn, the set of remaining proteins was found diluted.

**
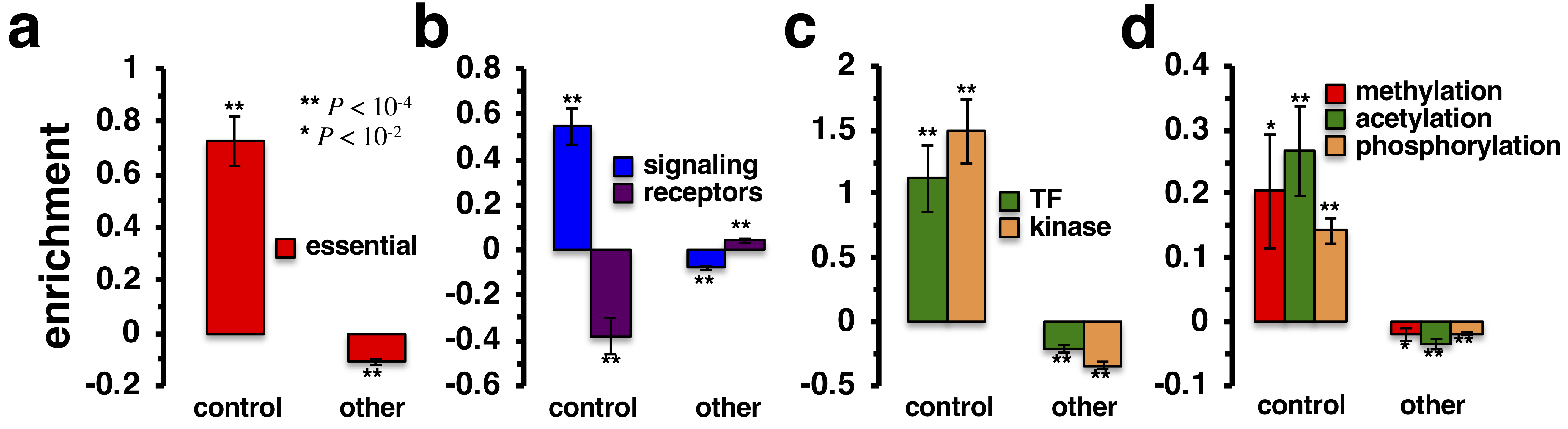
**

**Suppl. Figure 4.** Control proteins in pooled network of KEGG pathways. **(a)** Randomizing a set of essential genes, we observed that control genes in the network of combined KEGG pathways were significantly enriched, while remaining genes appeared diluted. **(b)** Similarly, signaling proteins excluding membrane bound proteins were significantly enriched with control genes while membrane bound proteins appeared diluted. **(c)** Furthermore, transcription factors and kinases predominantly appeared in the set of control genes. **(d)** As for posttranslational modifications, control proteins were enriched with methylated, acetylated and phosphorylated genes.

**
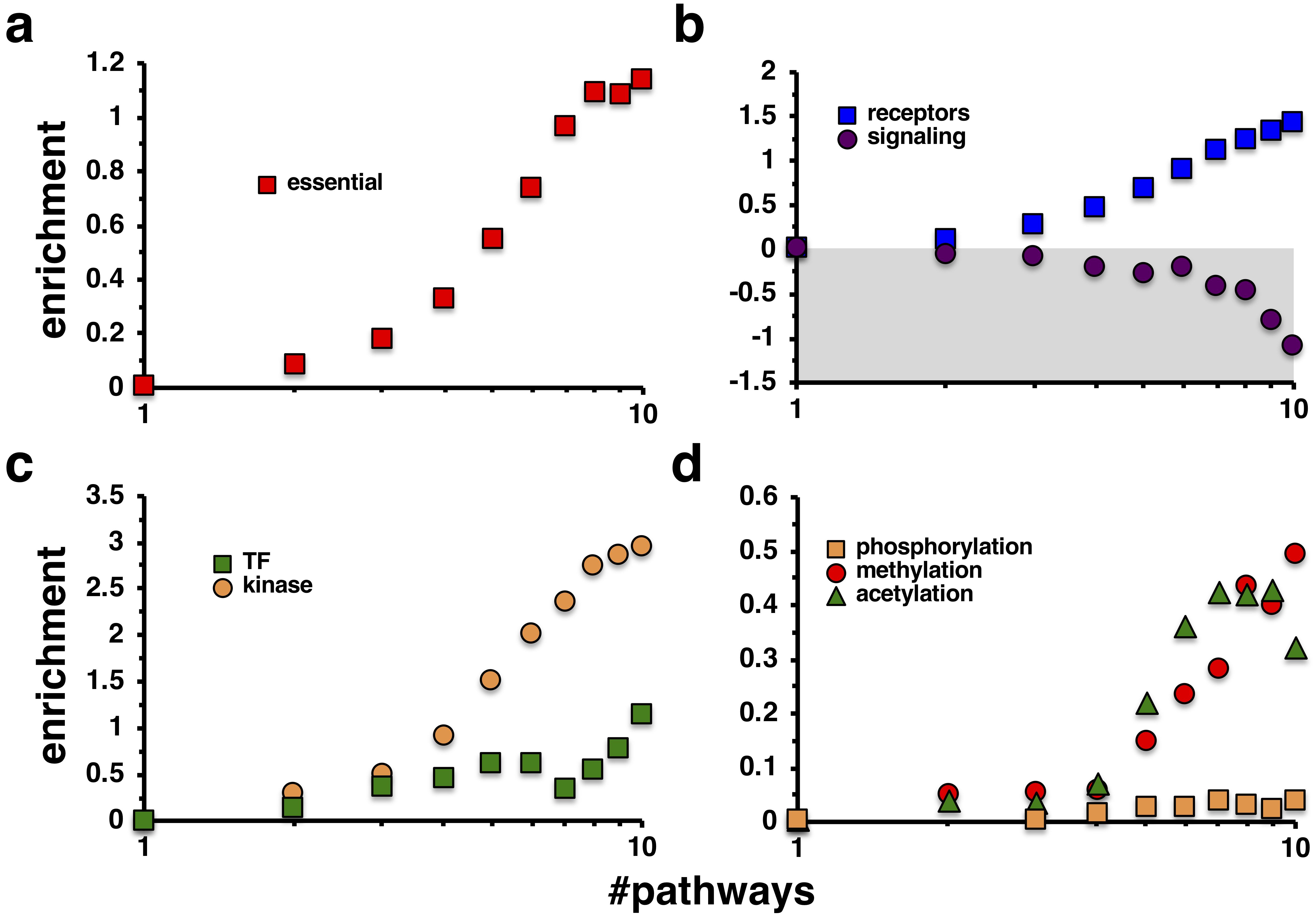
**

**Suppl. Figure 5.** Control proteins in Reactome pathways II. **(a)** Randomly sampling a set of essential genes, we found that essential proteins preferably were control proteins in an increasing number of pathways. **(b)** Similarly, membrane bound proteins were diluted among control proteins in an increasing number of pathways. In turn, signaling proteins excluding proteins with trans-membrane domain were enriched with control proteins. **(c)** Control proteins in an increasing number of pathways were more frequently found enriched with kinases than transcription factors. **(d)** Control proteins in a high number of pathways were strongly enriched with acetylated and methylated proteins while we found a modest enrichment of phosphorylated control proteins.

**
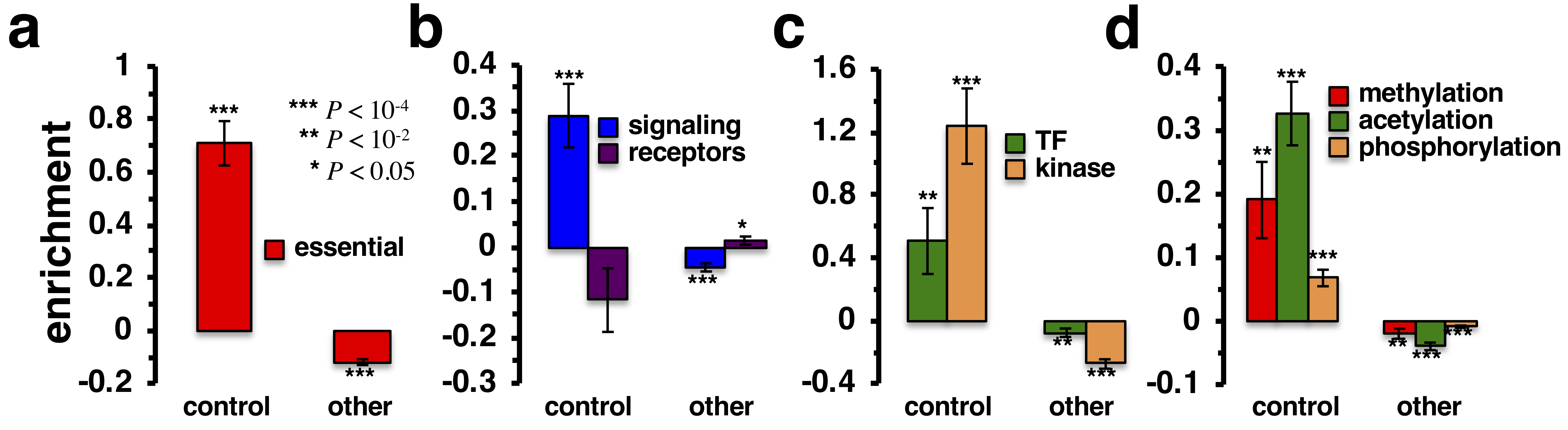
**

**Suppl. Figure 6.** Control proteins in a combined network of Reactome pathways. **(a)** Randomizing a set of essential genes, we observed that control genes in the network of combined Reactome pathways were significantly enriched, while non-control genes appeared diluted. **(b)** Similarly, signaling proteins excluding membrane bound proteins were significantly enriched with control genes. In turn, membrane bound proteins appeared diluted. **(c)** Furthermore, transcription factors and kinases predominantly appeared in the set of control genes. **(d)** As for posttranslational modifications, methylated, acetylated and phosphorylated genes are enriched with control genes as well.

**
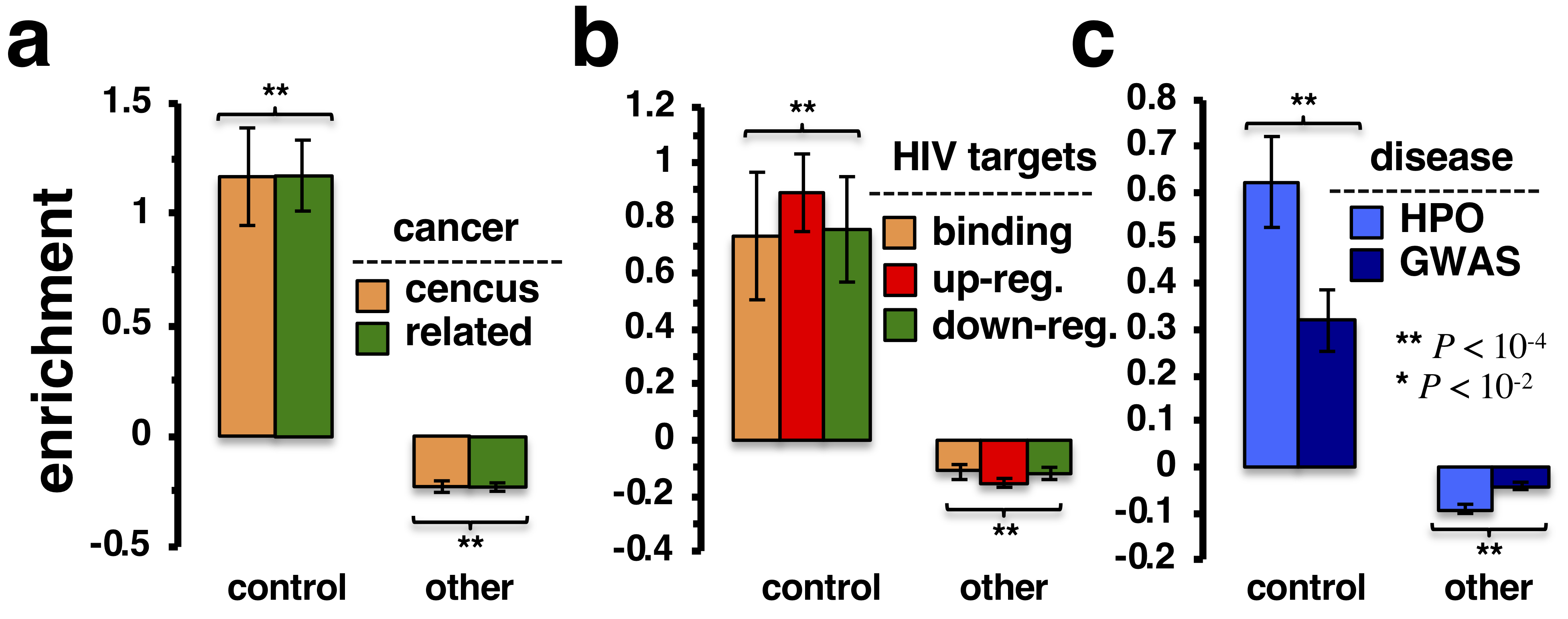
**

**Suppl. Figure 7.** Enrichment of control proteins in the combined KEGG pathway network with disease genes. **(a)** Randomizing a set of genes that were annotated as causally implicated in oncogenesis (census) and a set of onco- and tumorsuppressor genes (related), I found that control proteins in the combined KEGG pathway were enriched. **(b)** Similarly, control proteins harbor HIV virus targets and well as genes that were dys-regulated after viral infection. **(c)** Utilizing disease gene information from genetic (HPO) and genomic (GWAS) sources, I found that disease genes in general were preferably appearing as control proteins.


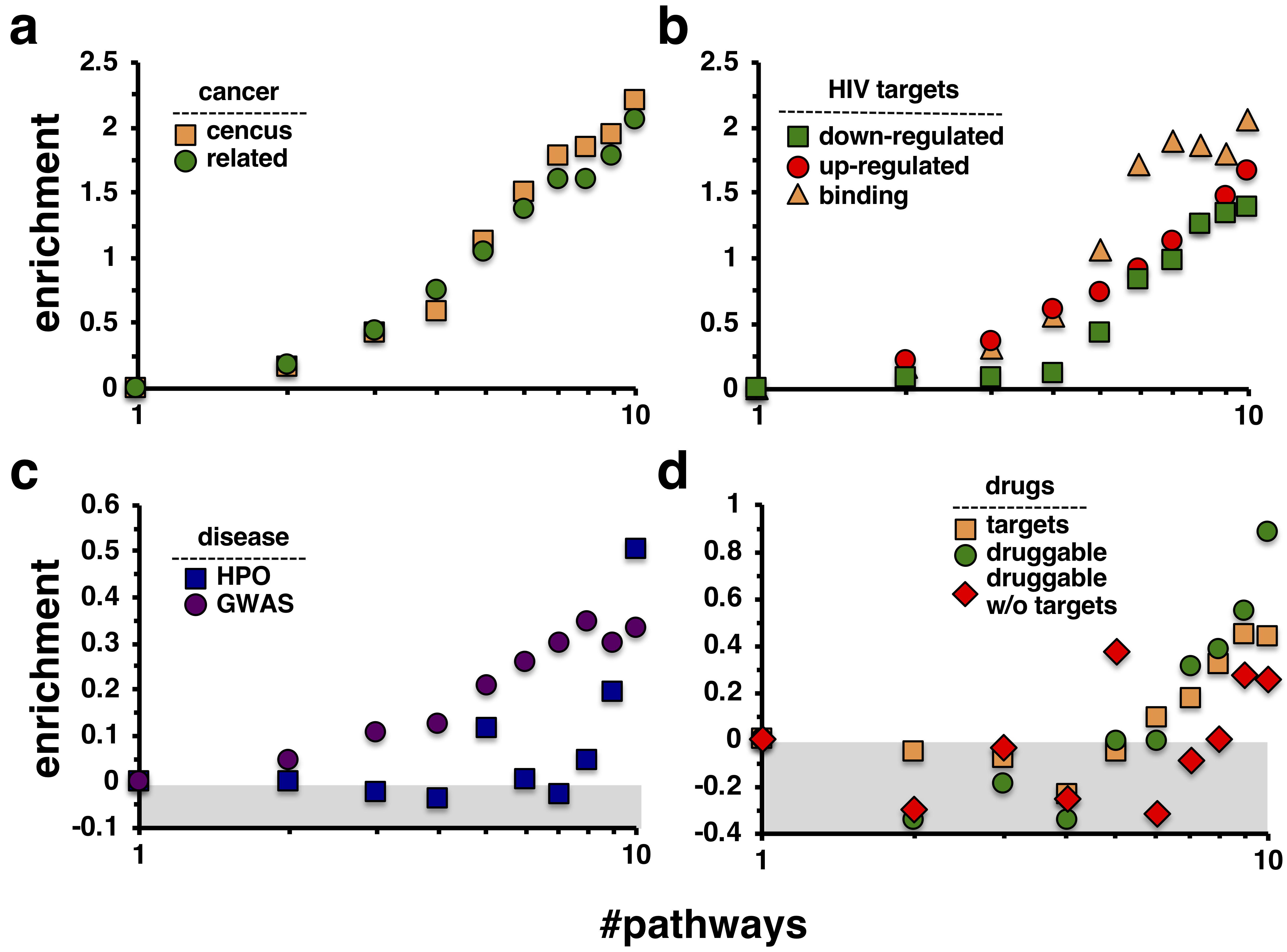


**Suppl. Figure 8.** Enrichment of disease genes and drug targets in groups of proteins that control different Reactome pathways. **(a)** Cancer genes strongly were enriched in bins of proteins that controlled an increasing number of Reactome pathways. **(b)** HIV virus targeted and dys-regulated genes upon infection appeared in such bins of control proteins as well. **(c)** Disease genes in general were frequently appearing as control proteins in an increasing number of pathways. **(d)** Drug targets and druggable genes were generally enriched in bins of proteins that control an increasing number of pathways.

**
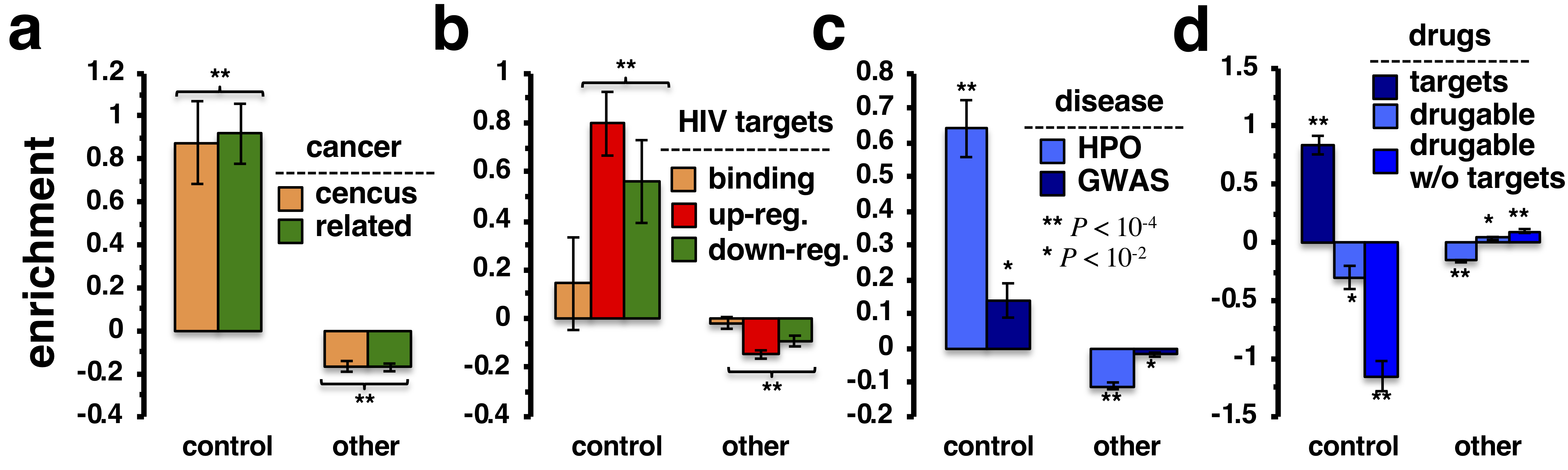
**

**Suppl. Figure 9.** Enrichment of disease genes and drug targets in proteins of the combined network of Reactome pathways. **(a)** Randomizing a set of genes that were annotated as causally implicated in oncogenesis (census) and a set of onco- and tumorsuppressor genes (related), I found that such proteins were enriched among proteins that controlled the combined network of Reactome pathways. **(b)** Similarly, HIV virus targeted control proteins and genes that were dys-regulated after viral infection appeared enriched with control proteins. **(c)** Utilizing disease gene information from genetic (HPO) and genomic (GWAS) sources, we found that disease genes in general were preferably appearing as control proteins. **(d)** Drug targets were found enriched with control genes, while we observed the opposite when we considered druggable genes.

**
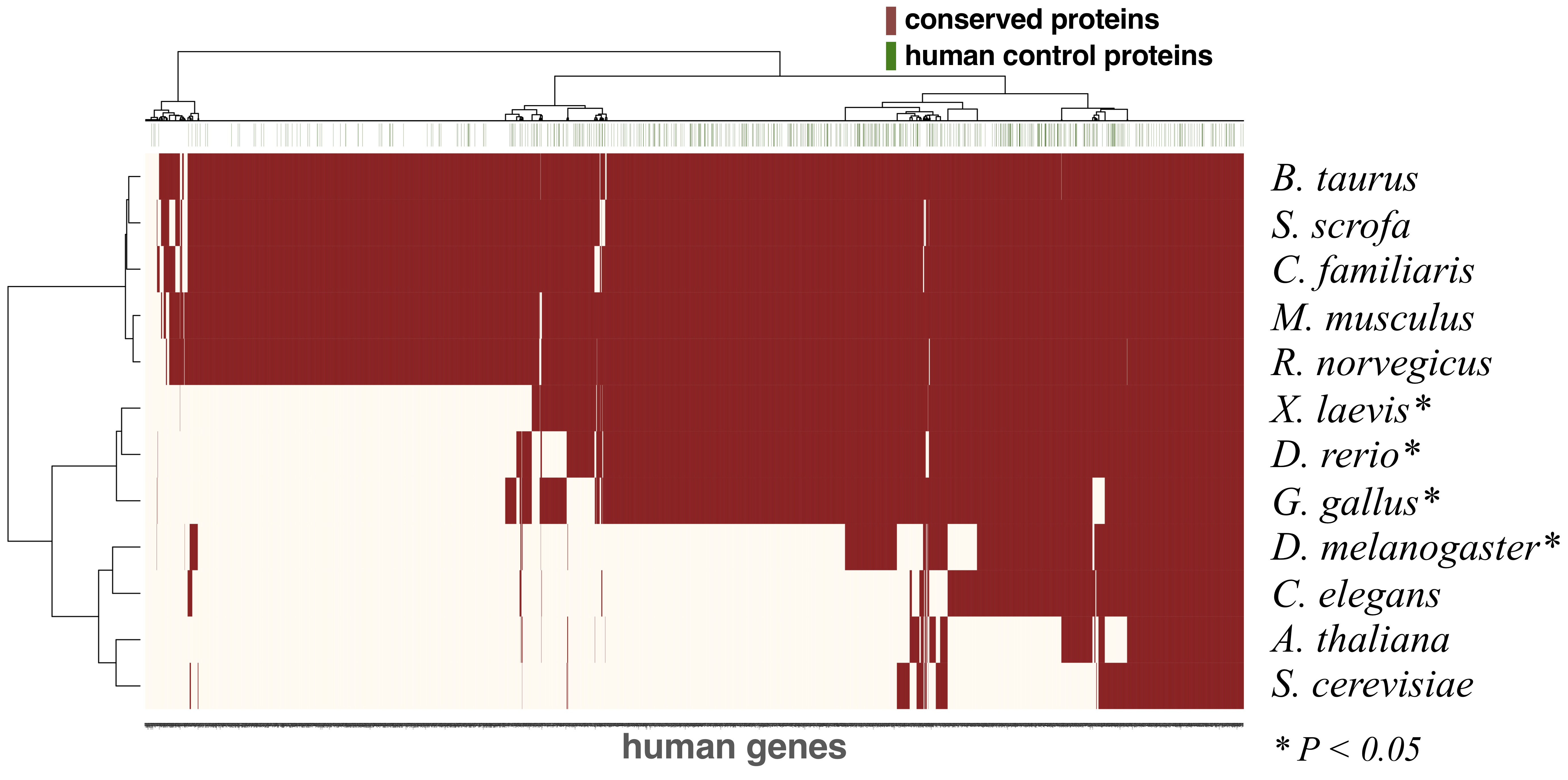
**

**Suppl. Figure 10.** Evolutionary conservation of human proteins. Using KEGG based groups of orthologs we labeled all human proteins in KEGG pathways with evolutionary conserved proteins in different organisms. Furthermore, we labeled all human control proteins that we found in the combined network of all human KEGG pathways.
